# Unveiling the Electronic Structure of Pseudotetragonal WO_3_ Thin Films

**DOI:** 10.1021/acs.jpclett.3c01546

**Published:** 2023-08-08

**Authors:** F. Mazzola, H. Hassani, D. Amoroso, S. K. Chaluvadi, J. Fujii, V. Polewczyk, P. Rajak, Max Koegler, R. Ciancio, B. Partoens, G. Rossi, I. Vobornik, P. Ghosez, P. Orgiani

**Affiliations:** † Department of Molecular Sciences and Nanosystems, Ca’ Foscari University of Venice, 30172 Venice, Italy; ‡ Istituto Officina dei Materiali (IOM)-CNR, Area Science Park, 34149 Trieste, Italy; § Theoretical Materials Physics, Q-MAT, CESAM, Université de Liège, B-4000 Liège, Belgium; ∥ Department of Physics, University of Antwerp, 2020 Antwerp, Belgium; ⊥ Area Science Park, Padriciano 99, 34149 Trieste, Italy; # University of Milano, I-20133 Milano, Italy

## Abstract

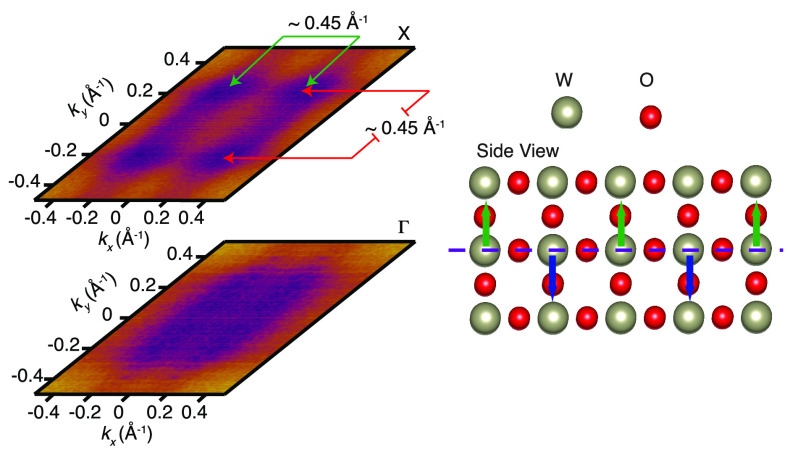

WO_3_ is a 5d compound that undergoes several structural transitions in its bulk form. Its versatility is well-documented, with a wide range of applications, such as flexopiezoelectricity, electrochromism, gating-induced phase transitions, and its ability to improve the performance of Li-based batteries. The synthesis of WO_3_ thin films holds promise in stabilizing electronic phases for practical applications. However, despite its potential, the electronic structure of this material remains experimentally unexplored. Furthermore, its thermal instability limits its use in certain technological devices. Here, we employ tensile strain to stabilize WO_3_ thin films, which we call the pseudotetragonal phase, and investigate its electronic structure using a combination of photoelectron spectroscopy and density functional theory calculations. This study reveals the Fermiology of the system, notably identifying significant energy splittings between different orbital manifolds arising from atomic distortions. These splittings, along with the system’s thermal stability, offer a potential avenue for controlling inter- and intraband scattering for electronic applications.

Controlling the electronic properties of quantum systems allows us to realize technological applications with improved performance, stability, and durability, as well as a significantly lower level of dissipation.^1−3^ This is particularly relevant for 5d-based transition metal oxides, which might provide a platform for integration into existing technology, with improved current densities, enhanced electrochromic and photovoltaic responses, and reduced switching energies.^4−12^ Therefore, understanding the electronic structure of quantum systems is a crucial task, especially for newly synthesized materials, and it allows us to pin down the hallmarks that describe their conductivity, their Fermi surfaces, and the relationship of the latter with symmetries and crystal structure. Among the 5d-based oxides, WO_3_ has been shown to be promising for applications, with the appearance of flexopiezoelectricity^10^ and electrochromism^11^ and as a realistic candidate for improving the performance of Li-based batteries.^13^ The range of applicability of this material extends also toward gas sensor applications,^14^ water splitting,^15^ memory devices,^16^ high-temperature diodes,^17,18^ and photodetectors.^19,20^ WO_3_ can be used to make faster and more efficient electronics,^4−12,21^ and it has been proposed theoretically as a candidate system for low-dissipation Rashba ferro- and antiferroelectrics.^22^ However, WO_3_ generally undergoes several different phase changes that make it difficult to be realistically used over a wide temperature range. Additionally, its electronic structure has not been experimentally investigated, although a few theoretical predictions have been reported.

Here, by using pulsed laser deposition (PLD),^23−26^ we exploit epitaxial strain to synthesize a thermally stable phase in thin films of the 5d compound WO_3_ (on a LaAlO_3_ substrate, LAO), and by using angle-resolved photoelectron spectroscopy (ARPES), we unveil the electronic structure and properties, which describe the Fermi surface. Here, we uncover the reference experimental benchmarks for the electronic band structure of WO_3_ thin films, which despite the numerous studies that rely on it^27−33^ is still lacking. In addition, by combining the experimental results with theoretical calculations, we report the existence of large distortion-induced band splitting, further enhanced by spin–orbit coupling (SOC), shedding light on the mechanisms by which orbital hybridization occurs.

WO_3_ thin films were grown by PLD at the NFFA facility.^25,26^ The growth was performed at ∼1000 K in an oxygen background pressure of 1 × 10^–3^ mbar (the typical deposition rate was 0.07 nm per laser shot). All of the investigated samples were grown on (001)-oriented LAO substrates. The ARPES measurements were performed *in situ* by using a Scienta DA30 hemispherical analyzer with energy and momentum resolutions better than 15 meV and 0.02 Å^–1^, respectively. The density functional theory (DFT) calculations were carried out within the CRYSTAL17 code^34^ based on a linear combination of localized basis functions and the B1-WC hybrid functional.^35^ To estimate/quantify the SOC, we also used the ABINIT code,^36,37^ as described in Methods.

The WO_3_ films were grown with thicknesses from 10 to 30 nm. We also used transmission electron microscopy (TEM) and X-ray diffraction to estimate the extent of relaxation as a function of thickness and also the surface roughness (see the Supporting Information). Within the range of thicknesses considered, we did not see by TEM any change in the lattice parameters or any change in the relaxation. By X-ray diffraction (XRD), the thinner films instead appeared to be flatter; therefore, we used these for the ARPES measurements (10 nm). Importantly, we also collected low-energy electron diffraction (LEED) (see the Supporting Information) both to monitor the quality of the surface and to look for possible surface reconstructions, which were not observed.

WO_3_ can be seen as an ABO_3_ cubic perovskite with a missing cation. It has, however, never been observed in the reference cubic structure, which exhibits various unstable phonon modes, including antipolar motion of W against O in various directions [X_5_^–^ and M_3_^–^ (see Figure 1a)] and oxygen octahedral rotations with different tilt patterns (M_3_^+^ and R_4_^+^).^30,38^ Accordingly, in the bulk form, WO_3_ undergoes several phase transitions as a function of temperature. Between 1300 and 1500 K, its structure is tetragonal (space groups *P*4/*nmm* and *P*4/*ncc*).^39,40^ At 1000 K it becomes orthorhombic (*Pbcn*),^35,39,40^ at room temperature monoclinic (*P*2_1_/*n*),^40,41^ and at 273 K triclinic (*P*1̅),^42^ and finally at 200 K, it enters a second monoclinic phase (*P*2_1_/*c*),^30,38,43,44^ with no further transitions down to 5 K. This implies that this monoclinic phase is the ground state of bulk WO_3_.^30^

**Figure 1:**
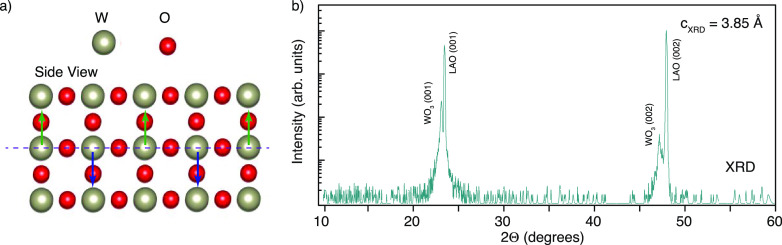
(a) WO_3_ tetragonal structure showing the out-of-plane opposite displacement of W and O atoms against one another. This phenomenon is also known as out-of-plane antipolar motion. (b) XRD θ–2θ scan of a WO_3_ film grown on LAO (film peaks are indeed identified by considering a tetragonal structure).

The lattice parameters of the room-temperature monoclinic *P*2_1_/*n* phase of bulk WO_3_ are as follows: *a* = 0.732 nm, *b* = 0.756 nm, and *c* = 0.772 nm.^40,41^ These remain very similar in the *Pbcn*, *P*1̅, and *P*2_1_/*c* phases. When the cell doubling in all three directions is taken into account, these lattice constants correspond to lattice spacings of ∼0.366 nm along *a*, ∼0.378 nm along *b*, and ∼0.386 nm along *c*. In the *P*4/*nmm* phase, the lattice spacing is instead ∼0.375 nm along *a* and *b* and ∼0.392 nm along *c*. With respect to the LAO substrate, characterized by an in-plane pseudocubic lattice parameter of 0.379 nm, an epitaxial tensile strain is therefore expected for all phases. In our work, the stabilization of a structural phase with a tetragonal metric at room temperature has been confirmed by XRD data of Figure 1b. From the (002) Bragg reflection, a *c*-axis parameter of 0.385 nm has been measured. The *c* value of this pseudotetragonal phase apparently matches that of the bulk *P*2_1_/*n* phase and other low-temperature bulk phases. This is, however, surprising in view of the tensile epitaxial strain conditions, expected to produce a significant contraction along *c*, and better suggests that our film could adopt a *P*4/*nmm* type of structure. It has nevertheless been shown that changing the oxygen pressure during PLD growth has a major impact on the film out-of-plane lattice constant.^32,45^

According to the report by Ning et al.,^45^ oxygen vacancies result in an increase in the *c* parameter of ≤5%. As in other oxides,^46,47^ oxygen vacancies appear to be preferentially located at specific positions of the perovskite structure rather being randomly distributed within the materials.^46−48^ The out-of-plane lattice expansion due to oxygen vacancies is often termed chemical strain.^49,50^ The measured *c* value of 0.385 nm obtained from our experiment is in very good agreement with the trend of the variation of the *c* lattice parameter with oxygen pressure reported in ref (45) for the *P*2_1_/*n* phase, suggesting that our pseudotetragonal film might in fact better adopt either that structure or that of the similar low-temperature phases.

To clarify this issue, we adopted an atomistic approach and performed DFT calculations. To determine the theoretical ground state of the WO_3_ film, we focused on the six phases that are observed experimentally in the sequence of structural phase transitions of bulk WO_3_^38−44^ and explored their energy gain under tensile strain. Starting from the atomic positions of their fully relaxed bulk structures, we fixed their *a* and *b* lattice parameters to the pseudocubic *a*_LAO_ of 0.379 nm while relaxing the *c* parameter. Our calculations suggest that the theoretical ground state of the film should be the strained monoclinic *P*2_1_/*n* phase with a *c* parameter of 0.738 nm. This result is in line with previous studies of stoichiometric WO_3_ films, for which the *c* parameter was measured to be 0.733 nm^45,51,52^ and the structure of the film identified as being similar to that of the monoclinic *P*2_1_/*n* phase.^45^ This result is, however, questioned by the observed *c* parameter of 0.77 nm in our XRD.

As previously discussed, our films grown at a low oxygen pressure are deficient in oxygen. This was further confirmed by our photoemission data, which report metallic character for the samples, with the Fermi level crossing the conduction band, instead of an insulating behavior expected for the stoichiometric phase of WO_3_. The oxygen vacancies then give rise to a chemical strain, artificially increasing the *c* parameter. Following what was done in ref (50), the substoichiometric character of our WO_3_ film was then simulated by treating oxygen vacancies as a strain constraint in the out-of-plane direction. Accordingly, in addition to the relationship *a* = *b* = *a*_LAO_, the *c* parameter was fixed to the experimental value (*c* = 0.77 nm). The energy gain diagram presented in Figure 2a indicates that in this specific case, the most stable configuration corresponds to the *Pbcn* structure. This suggests that our pseudotetragonal films might likely adopt that structure, which will be further confirmed later via inspection of the electronic properties.

**Figure 2:**
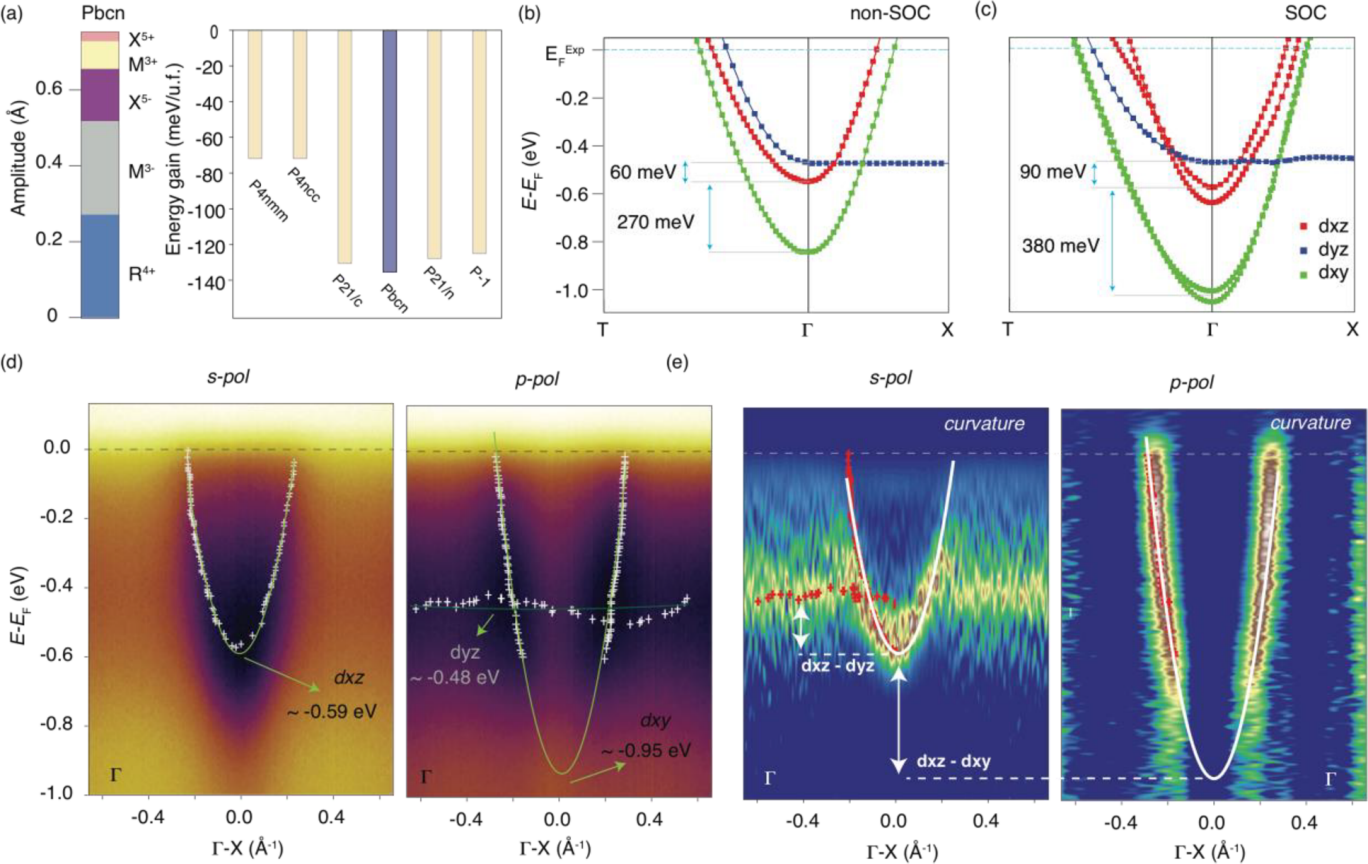
(a) Symmetry-adapted mode decomposition pseudotetragonal thin film with the *Pbcn* strained structure of WO_3_ (left) and the DFT-calculated energy gain of the six phases that are observed experimentally in the sequence of structural phase transitions of bulk WO_3_ with respect to the cubic phase (right), obtained by fixing the lattice parameters to the experimental ones to take into account the tensile strain as well as the strain constraint in the out-of-plane direction to account for the substoichiometric nature of our WO_3_ film. The DFT electronic structure of a pseudotetragonal thin film with the *Pbcn* strained structure is shown in panels b and c without and with SOC, respectively. The relevant energy splitting is captured by DFT, and the orbital mixing induced by SOC further enhances the energy separation. The Fermi level in the DFT calculations was aligned with the experimental value by rigidly shifting the calculated bands. As shown in Figure S3, where the electronic structures of the similar low-temperature strained phases of WO_3_ are presented, our ARPES data are compatible with only the strained *Pbcn* structure. Only in this structure are the orbitals in the same sequence, and the d_*xz*_ and d_*xy*_ orbitals are split considerably. (d) ARPES data along the Γ–X direction are shown, showing good agreement with the calculations. The minima of the d_*yz*_ and d_*xy*_ bands are shown, as well as an average value for the energy at which the dispersionless d_*xz*_ orbital is located. (e) ARPES curvature plots for better visualization of the energy states and their relative separation, indicated by the white arrows for the bands relative to each other’s.

By using AMPLIMODE software,^53^ we performed symmetry-adapted mode analysis to identify the distortions, which play a major role in the stabilization of such a *Pbcn* strained phase. It can be characterized (see Figure 2a) by (i) octahedral rotations (R_4_^+^ and M_3_^+^ modes) with tilt pattern *a*^0^*b*^+^*c*^–^ in Glazer’s notation,^54^ (ii) an antipolar motion along *y* (X_5_^–^ mode), (iii) a small contribution of a bending mode (X_5_^+^), and (iv) an antipolar motion along the *z*- and *x*-axes (M_3_^–^ mode), where the *x* component of the M_3_^–^ mode appears through anharmonic coupling.^38^ This is in contrast with the *P*2_1_/*c* ground state of bulk WO_3_ that arises from the contributions of (i) R_4_^+^ with tilt pattern *a*^–^*a*^–^*c*^–^, (ii) antipolar motion along the *z*-axis (M_3_^–^), and (iii) antipolar motion with the same amplitude along the *x*- and *y*-axes (X_5_^–^).^55^

Remarkably, we note that the pseudotetragonal thin films are incredibly resilient and their structure survives within a large temperature range, i.e., from room temperature (as demonstrated by XRD) to, at least, 77 K (as confirmed by ARPES). This indicates that WO_3_ on LAO is highly structurally and thermally stable and that the substrate can freeze the overgrown thin layers and make them robust against temperature variations. This is in contrast to the bulk behavior, in which orthorhombic (or tetragonal) phases have never been found at low temperatures but only at temperatures as high as 800 K.^39−41,56,57^ Again, this result points to the importance of epitaxial strain in realizing films with enhanced thermal stability compared to that of their bulk counterpart.

To understand the role of the crystal structure in the electronic properties of this compound, we performed ARPES with in-vacuum transfer without exposing the samples to air. First, we notice that the tetragonal metric of the WO_3_ films is also reflected in the symmetries of the reciprocal space, namely in the symmetry of the Fermi surface (see Figure 3a). The latter shows a Fermi level crossing along *k*_*x*_ and *k*_*y*_ of 0.45 Å^–1^, as indicated in Figure 3a. From the Γ to X points of the Brillouin zone (BZ) (Figure 3a), we did not observe any appreciable change in the Fermi surface volume within our experimental resolution range; however, an overall different shape is visible, as expected for this system, which electronically speaking still behaves as a bulklike system for the ARPES probing depth. To locate the high-symmetry positions along the *k*_*z*_ direction, we performed photon energy-dependent scans (see the Supporting Information for a plot of *k*_*z*_ vs photon energy dependence), and we show them in Figure 3b. Here, we see “hot spots” of spectral intensity at several *k*_*z*_ values. This repeating behavior helps us to fix the *k*_*z*_ corresponding to the high-symmetry points of the Brillouin zone, namely, X and Γ, and allows us to make an estimate of the *c*-axis from ARPES. With an inner potential of 11 ± 3 eV, we obtain a *c*_ARPES_ of 0.77 nm, in agreement with the results from XRD. We notice that ARPES gives exactly twice the XRD value, indicating that the unit cell has a doubling, here revealed by a resonant behavior of the spectra, which reflects in this case the major probability of initial–final state matching in the photoemission process for states excited from the Γ and X points. The structural distortions of the pseudotetragonal phase lead to large energy splittings, in contrast to the *Pbcn*, *P*2_1_/*n*, and *P*2_1_/*c* bulk phases (see Figure S2). In high-temperature tetragonal bulk WO_3_, the d_*xz*_ and d_*yz*_ orbitals are degenerate at the center of the BZ (see Figure S2a). However, in the pseudotetragonal thin film, the orbital degeneracy is removed, resulting in an energy splitting that can be resolved by ARPES measurements and takes the experimental value of 100 meV (Figure 2d,e). This splitting is consistent with our calculations in the strained *Pbcn* phase, although the computed value takes a smaller value of 60 meV (see Figure 2b).

**Figure 3:**
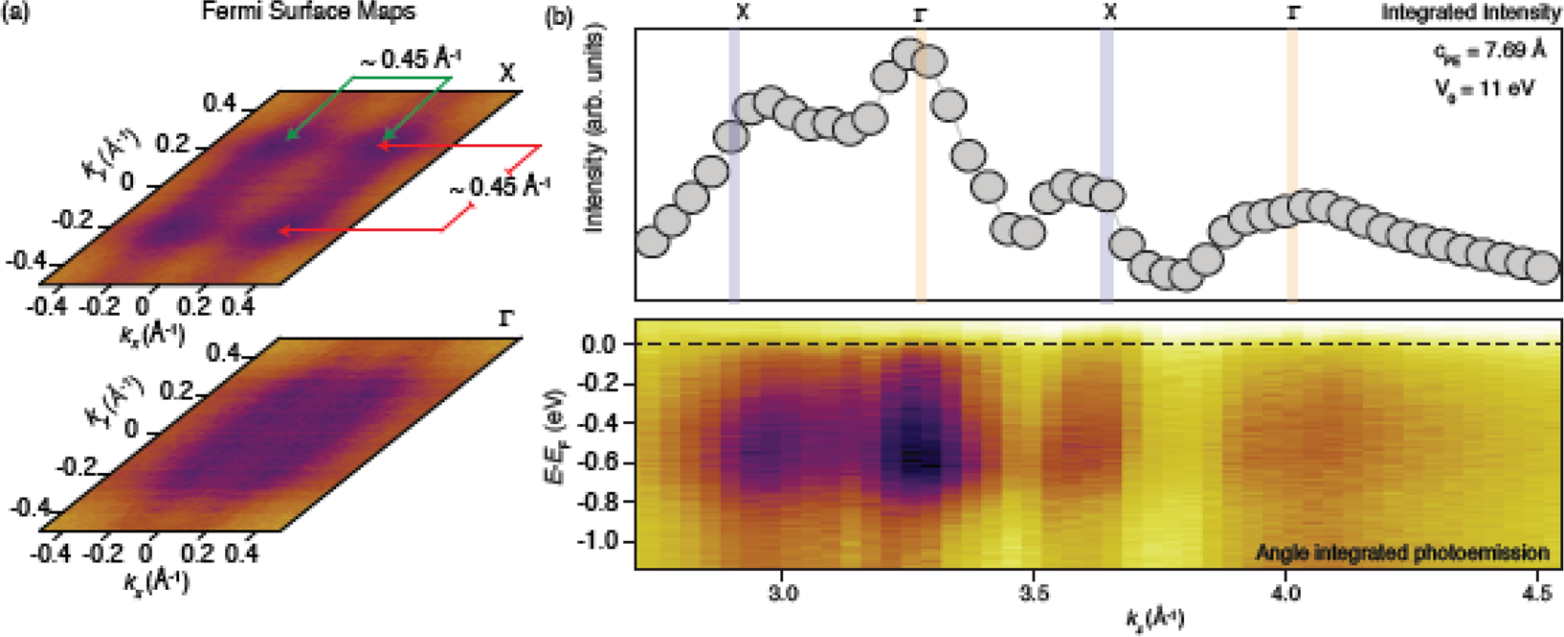
(a) Fermi surfaces collected for the first X and Γ points in panel b. The pattern observed in the spectra is consistent with a tetragonal unit cell structure. For reference, we have also shown the calculated isoenergetic cuts across the WO_3_ conduction band, which show a good agreement with the experiment (Figure S4). (b) Photon energy scans and resonances of the material. The inner potential used (*V*_0_) was 11 eV. The procedure is explained in the Supporting Information.

Upon inclusion of the SOC, which is expected to be relevant for 5d orbitals, this discrepancy finds a solution; our calculation in the strained *Pbcn* phase reproduces an energy splitting of ≈90 meV between the d_*yz*_ and d_*xz*_ orbitals, in perfect agreement with ARPES (Figure 2c). This emphasizes the SOC’s critical role in hybridizing the orbitals in 5d oxides. However, our results suggest that the effect of structural distortions is greater than that of the SOC. In the WO_3_ film, the amplitude of the antipolar distortion along *y* is greater than along *x*; as a result, the upshift of the d_*yz*_ energy level is larger than that of the d_*xz*_ energy level. This is because an increase in the *y*-direction antipolar distortion results in a stronger overlap between the O 2p_*y*_ and W 5d_*yz*_ orbitals,^54^ which in turn causes an upshift in the related antibonding energy level. Thus, the splitting between the d_*yz*_ and d_*xz*_ orbitals in this pseudotetragonal phase (shown in Figure 2b,c) is caused by a proper balance between the amplitude of the antipolar motions along *y* (X_5_^–^ mode) and *x* (M_3_^–^ mode). Note that this splitting, with the d_*yz*_ orbital located at an energy level higher than that of the d_*xz*_ orbital, is absent in all of the bulk phases, including the *Pbcn* and *P*2_1_/*n* phases where the *x* component of the M_3_^–^ mode is negligible, or in the bulk form of the ground state where the amplitudes of the antipolar motion along *y* and *x* are almost the same (see Figure S2). More importantly, as shown in Figure S3, this is not the case in any of the similar low-temperature phases under strain, providing additional evidence that our pseudotetragonal film adopts a strained *Pbcn* structure.

A second splitting can also be observed in the DFT results between the d_*xz*_ and d_*xy*_ orbitals, and it is estimated to be ≈380 meV after inclusion of SOC (see Figure 2c). Our calculations indicate that octahedral tilting (M_3_^+^ and R_4_^+^ modes) with deviations of the W–O–W angle from 180° also involves tuning the overlap of orbitals in this case.^58^ From ARPES (Figure 2d,e), it is more challenging to make a straightforward comparison with the DFT results, because the d_*xy*_ band has strong matrix elements that suppress its intensity near the center of the BZ.^58,59^ The matrix elements and the fact that varying the probe polarization vector allows us to measure different orbital contributions are well-known among the photoemission community and are described in a dedicated section of the Supporting Information. Despite the matrix elements, we can extrapolate the minimum by fitting the data, obtaining a d_*xz*_–d_*xy*_ separation of ≈400 meV, which is also in close agreement with the calculated value. Thus, the structural distortions are very important in defining the electronic properties of WO_3_, and the strain is crucial for stabilizing the pseudotetragonal phase observed here.

In conclusion, we report the existence of a new phase in WO_3_, which we call a pseudotetragonal phase but reveals in fact a strained *Pbcn* phase. This phase observed in films grown at low oxygen pressures differs from the *P*2_1_/*n* phase previously reported in stoichiometric films. It accommodates antipolar distortions along all three axes. Such distortions are important for understanding the vibrational modes and the electronic properties of this system. By combining XRD, TEM, DFT calculations, and ARPES, we determine the role and consequences of the structural distortions on the WO_3_ electronic structure, experimentally revealing band splittings as large as 400 meV between the d_*xz*_ and d_*xy*_ orbitals and 100 meV between the d_*yz*_ and d_*xz*_ orbitals, reminiscent of the proper balance between the amplitude of the M_3_^–^ and X_5_^–^ antipolar modes in different directions.^30,38,59−61^ Finally, we show a large thermal stability for the grown films, and we demonstrate that SOC plays a sizable role in the interpretation of the electronic behavior of WO_3_. Our work not only motivates the use of strain to realize novel structural phases in binary 5d oxides but also shows us how to use it to tune their orbital degrees of freedom.

## Methods

*DFT Details*. To approximate the BZ, integration over 8 × 8 × 8 k-point meshes for the cubic symmetry or meshes with equivalent sampling for other phases (e.g., meshes of 6 × 6 × 8, 6 × 6 × 4, and 4 × 4 × 4 for the *P*4/*nmm*, *P*4/*ncc*, and *Pbcn* phsases, respectively) were used. In the CRYSTAL17 code,^33^ the self-consistent-field (SCF) convergence’s tolerance of the change in total energy was set to 10^–10^ Hartrees. Geometry optimization was performed by employing a quasi-Newton approach with a BFGS Hessian scheme, so that a specific space group symmetry was preserved for each structure during the structural relaxations. The root-mean-square values of the gradient and displacements were converged to <5 × 10^–5^ Hartrees/Bohr and 10^–3^ Bohr, respectively. We also used the ABINIT code^36,37^ with a plane-wave basis set and the LDA functional with Perdew–Wang’s parametrization,^62^ to include SOC for the electronic band structures. In this case, the electronic wave functions were expanded in plane waves up to an energy cutoff of 60 Hartrees, and the electronic self-consistent calculations were converged until the difference in the total energy is <10^–9^ Hartrees.

## Supporting Information Available

Additional ARPES spectra and calculations for other phases, experimental setup and geometry, and a discussion of the matrix elements (PDF)
