# Correction to “Unveiling the Electronic Structure of Pseudotetragonal WO_3_ Thin Films”

**DOI:** 10.1021/acs.jpclett.3c02358

**Published:** 2023-09-05

**Authors:** F. Mazzola, H. Hassani, D. Amoroso, S. K. Chaluvadi, J. Fujii, V. Polewczyk, P. Rajak, Max Koegler, R. Ciancio, B. Partoens, G. Rossi, I. Vobornik, P. Ghosez, P. Orgiani

F. Mazzola (federico.mazzola@unive.it) and P. Orgiani (pasquale.orgiani@cnr.it) are co-corresponding authors.

