# Supplementary material for: Unveiling the Electronic Structure of Pseudotetragonal WO3 Thin Films
Source: J Phys Chem Lett. 2023 Aug 8;14(32):7208–14. doi: 10.1021/acs.jpclett.3c01546 (PMC10440808; doi:10.1021/acs.jpclett.3c01546)
Supplement: Supplementary file 1 — jz3c01546_si_001.pdf [file jz3c01546_si_001.pdf]

Supplementary Information: Unveiling the Electronic Structure of Pseudo-Tetragonal WO_3 Thin Films

F. Mazzola,[1, 2, 3,+, *] H. Hassani,[4, 5, +] D. Amoroso,[4] S.K. Chaluvadi,[1] J. Fujii,[1] V. Polewczyk,[1] P. Rajak,[1]Max Koegler,[1] R. Ciancio,[1] B. Partoens,[5] G. Rossi,[6, 1] I. Vobornik,[1] P. Ghosez,[4] and P. Orgiani[1]

[1]*CNR-IOM TASC Laboratory, Area Science Park, I-34149 Trieste, Italy*
[2]*Department of Molecular Sciences and Nanosystems, Ca Foscari University of Venice, 30172 Venice, Italy*
[3]*Istituto Officina dei Materiali (IOM)-CNR, Laboratorio TASC,Area Science Park, S.S.14, Km 163.5, 34149 Trieste, Italy*
[4]*Theoretical Materials Physics, Q-MAT, CESAM, Université de Liège, B-4000 Lige, Belgium* [5]*Department of Physics, University of Antwerp, Groenenborgerlaan 171, 2020 Antwerp, Belgium*[6]*University of Milano, I-20133 Milano, Italy*

+ These authors contributed equally to this work

* federico.mazzola@unive.it

ARPES k_z plot procedure and measurements

In order to access the electronic dispersion along the k_z direction, as shown in Fig. 3b of the main text, we collected ARPES energy-k dispersions at several photon energies. Being the photon energy and the k_z directly proportional, we can readily obtain the dispersion along the out-of-plane direction of the Brillouin zone. In particular:

$$k_z = \frac{1}{\hbar}\sqrt{2m(E_{kin}cos^2\theta + V_0)} \quad (1)$$

where, m is the electron mass, E_{Kin} the electrons' kinetic energy, θ is the polar angle, and V_0 is the inner potential. Thus, when varying photon energy, one is able to detect the electronic structure of solid state systems along the k_z direction by verifying how the bands change. As an example, we show the linear vertical polarization data for WO_3 collected for some photon energies in Fig.1.

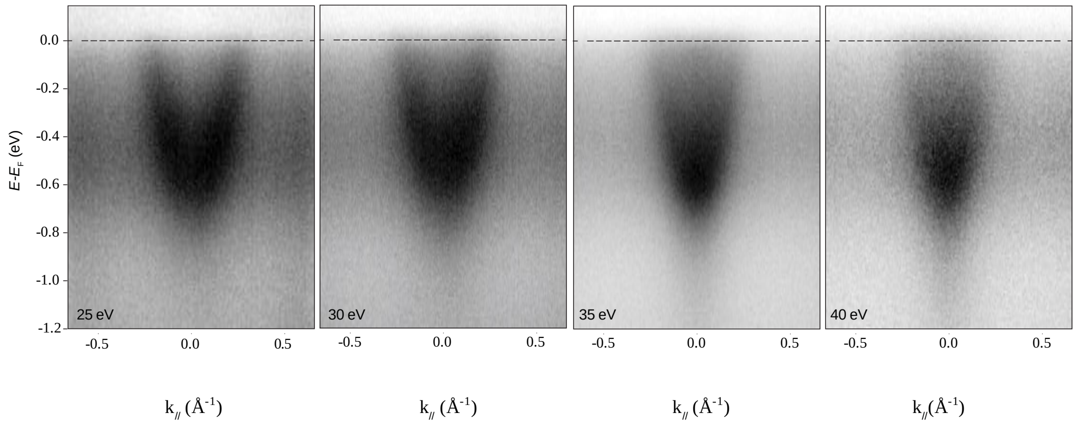

FIG. S1. Electronic structure of WO3 collected by ARPES at various photon energies, showing changes which can be attributed to a combination of matrix elements and to the three-dimensional character of the material. These plots relate directly to figure 3b of the main text.

We notice that the ARPES measurements were possible because the samples had states at the Fermi level. This is a known situation which occurs for many oxides, in which even a mild chance in oxygen balance could affect the electronic properties, without changing necessarily the structure [1, 2].

From data collected at different photon energy, we can also extract the location in kz of the high-symmetry points, as in Fig. 3 of the main text. What we have done in the current case is the following: we extracted from each ARPES map an energy distribution curve (EDC) at k=0 and we plot its intensity as function of both function of binding energy and photon energy. The result of this procedure, which gives the plot in Fig. 3b of the main text shows some resonances which are indicative of the most favorable initial-final state matching and that can be used to extract the high symmetry points in the out of plane direction of the Brillouin zone. By taking into account of a c-axis of 0.77 nm and an inner potential of V=11±3 eV we found the best repetition fit the zone with the data. The best fit is obtained by simultaneously accounting for V and c. V does not change much the conclusions if taken from 8 to 14 eV.

ARPES fitting details

The ARPES spectra have been fitted by using Lorentzian curves convoluted by a Gaussian to account for the resolutions of the instrument, which were better than 15 meV and 0.02 $^{-1}$, for energy and momentum respectively. The data were fitted by using energy-distribution and momentum-distribution curves to properly account for the curvature of the various features: for example, at the minimum of the parabolic dispersions, energy distribution curves capture better the energy dispersion. At the Fermi level, instead, momentum distribution curves are more suited to give an accurate determination of the k-position of the electronic states.

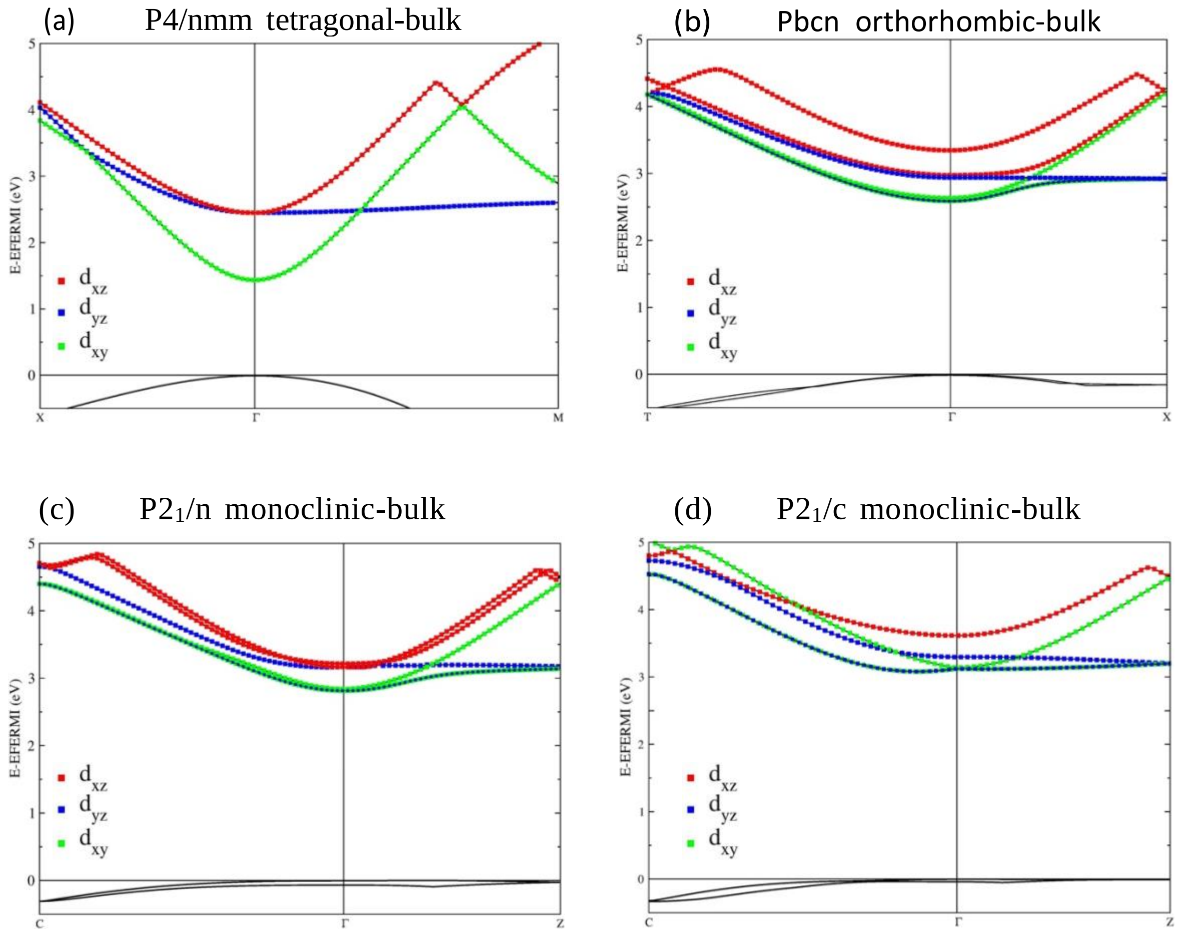

FIG. S2. (a) DFT electronic structure of the P4/nmm tetragonal phase in the bulk form, (b) the orthorhombic Pbcn phase, (c) the room-temperature $P2_1/n$ monoclinic phase, and (d) the ground state $P2_1/c$ monoclinic phase. The large splittings in the WO_3 thin films, with the same electronic level arrangement, are absent in all the phases of bulk form.

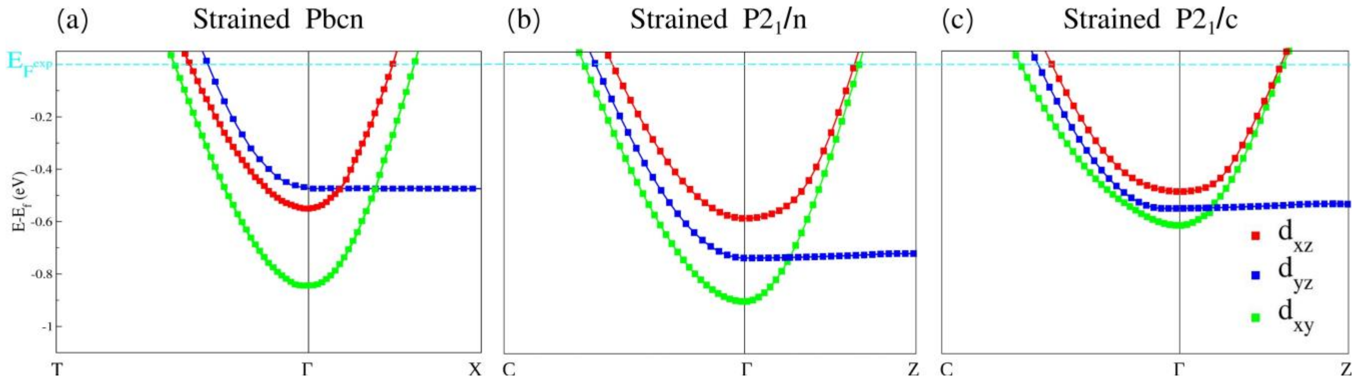

FIG. S3. DFT electronic structure (without SOC) of the pseudo-tetragonal thin film with (a) strained Pbcn structure, (b) strained $P2_1/n$ structure, and (c) strained $P2_1/c$ structure. The Fermi level in the DFT calculations has been aligned to the experimental value by rigidly shifting the calculated bands. It can be seen that our ARPES data are only compatible with strained Pbcn structure. Only in this structure are the orbitals in the same sequence, as well as the large splitting of d_{xz} and d_{xy}, which is reminiscent of the correct balance of the amplitude

of the M_3^- and X_5^- antipolar modes in various directions.

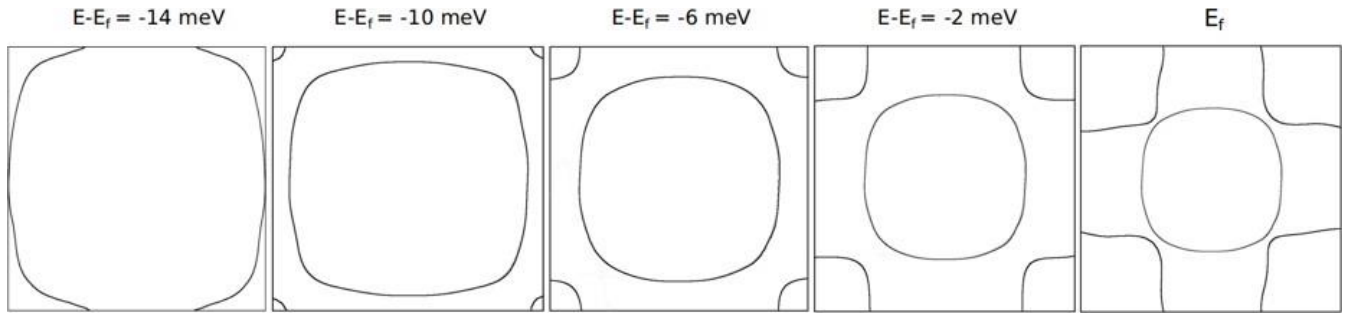

FIG. S4. DFT calculated iso-energetic cuts, showing a good agreement with the experimental Fermi surface in Fig. 3 (a) of the main text (The E_f panel can be used for the comparison).

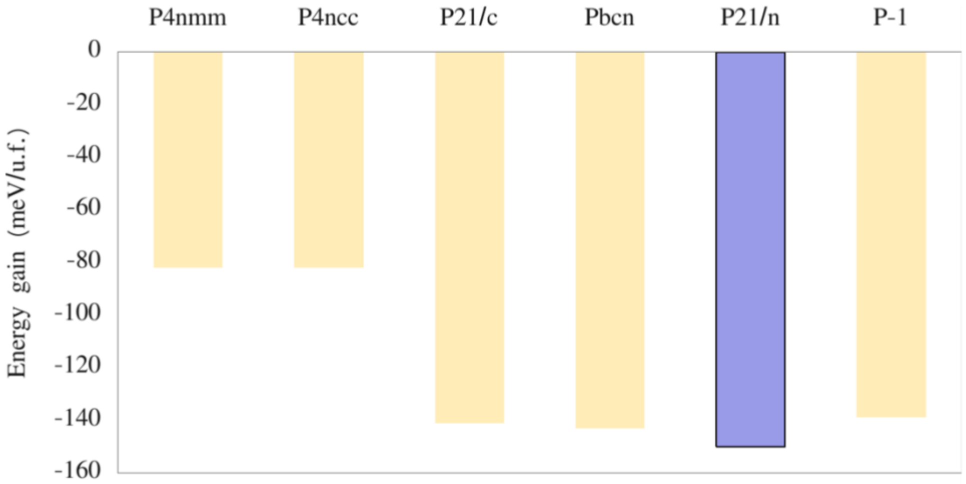

FIG. S5. The DFT calculated energy gain of the six phases of thin films WO_3 with respect to the cubic phase, obtained by fixing a and b lattice parameters to the pseudo-cubic LAO = 0.379 nm, while relaxing the c parameter. Our calculations suggest that the theoretical ground state of the film should be then the strained monoclinic P21/n phase with c = 0.738 nm.

Transmission electron microscopy (TEM) data and Low energy electron diffraction (LEED)

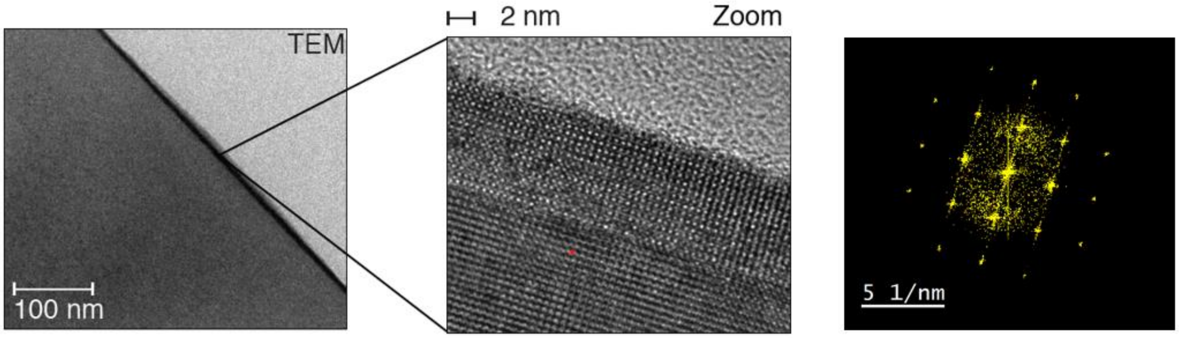

FIG. S6. TEM image showing a very sharp interface between the substrate LAO and the overgrown film. To the right, the zoom show no interfacial defects and no sign of relaxation. To the right, TEM fast Fourier transform showing a “pseudo”-cubic perovskite structure

The quality of the films is also assessed by a transmission electron microscopy (TEM) investigation which reveals with atomic resolution a very sharp interface between the WO3 films and the LAO substrate with no presence of spurious phases (e.g. randomly oriented phases, minority phases). In addition, the structure of the film over the whole image is homogeneous, and free of significant defects. No structural differences were detected among the near-interface region and the bulk of the film, as well as no traces of spurious phases or segregation of crystalline phases other than WO_3. The Fast Fourier Transformation (FFT) patterns calculated from the film region can be safely assigned to a “pseudo”-cubic perovskite structure (FFT are not sufficiently precise to determine

the in-plane as well as the out-of-plane lattice parameters as XRD).

This is compatible with a tetragonal structure with WO3 in-plane lattice parameters matching those of LAO substrate of 0.379nm and the out-of-plane lattice parameter equal to 0.385 nm – value also extracted by symmetrical theta-2theta scan. with the absence of superstructure reflections indicating an orthorhombic structure.

To ascertain the quality of the sample and the absence of possible surface reconstructions, we performed LEED. The latter, at room temperature as the XRD hint at the presence of the same. Within the experimental resolutions, we did not observe any appreciable change in the phase of the material probed by the various techniques and the spots of the LEED do not indicate any overgrown structural phase.

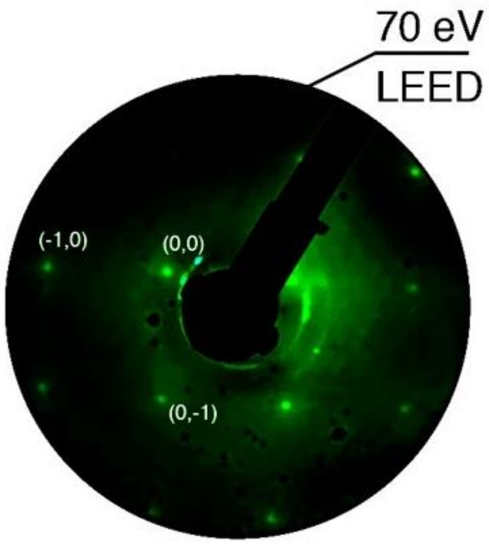

FIG.S7 WO_3 LEED at room temperature with no sign of in-plane superstructure.

ARPES details

(a) Geometry

The current section describes experimental details useful to better understand the ARPES data presented in the main text. First of all, let us show the experimental geometry, which has an important influence on the photoemission matrix elements on which the visualization of the corresponding bands is based. This is also particularly important when one uses polarized synchrotron radiation to as a probe. As in FIG. 9, our experiment was such that linear vertical light is fully in the plane of the sample, while linear horizontal is 50% in plane and 50% out of plane.

In our work, we performed ARPES by using both linearly polarized light, both linear horizontal and vertical. The

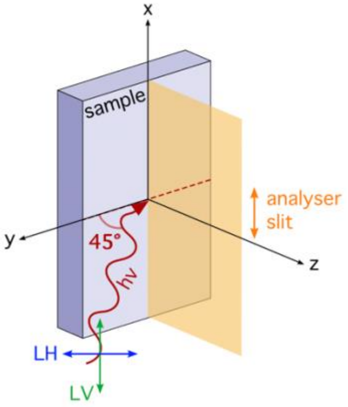

FIG. S8 ARPES geometrical setup and light polarization arrangement.

former, with respect to the sample mirror plane, couples to both odd and even components of the orbitals (with equal contribution), while the latter only to the even components. This results in selection rules which highlights the orbitals with the corresponding non-zero contributions. In particular, linear vertical couples mostly to the dxz orbitals, whilst linear horizontal better highlights the dyz and dxy. This strategy has been used to get an overall visualization of the electronic bands.

(b) Broadening

Another important peculiarity of ARPES is the ability to reveal the lifetime of the quasiparticles, often exemplified by the width of the bands. In the present case, as one can notice, the ARPES spectra appear quite broad. Generally, the broadening is very material-dependent but this can be expected to be given by various factors: if the bands are finely split by spin orbit (like in this case) but their separation is very small, the resolution will smear them significantly. There are other factors which might contribute to the broadening of the bands, namely the many body interactions. For systems which are 3D and with a large orbital overlap along the z direction, which is important for 5d compounds, kz will certainly contribute to the broadening. We simulate here, as an example what can happen, with a kz broadening. In particular, we simulated a parabolic dispersion and we made it disperse with kz in a realistic way (different color corresponds to a different kz, with 250 meV dispersion, so we kept it even quite small). We included 100 meV of impurity-electron scattering (which is also a small value similar to high quality graphene on SiC to be on a safe side) Then we convoluted them by the experimental resolutions (~12 meV and 0.018 1/Å). After we included a decay in the intensity from the red curve to the upper ones which decreases it exponentially I~I0*exp(-kz). We assumed (which is reasonable for the mean free path of the energy used in our experiment and the c axis of our system, 1/3 of a BZ). As one sees, the resulting ARPES is significantly broader and this is not related to sample quality.

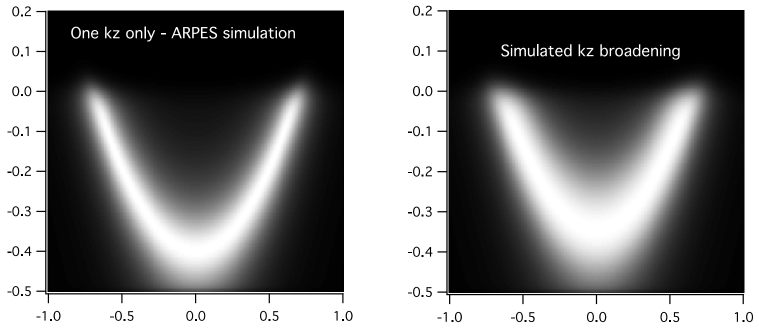

FIG. S9 ARPES simulation of bands without (left) and with (right) kz broadening. Note that a finite resolution has been included in both.

References:

(1) Raj, S.; Matsui, H.; Souma, S.; Sato, T.; Takahashi, T.; Chakraborty, A.; Sarma, D. D.; Mahadevan, P.; Oishi, S.; McCarroll, W. H.; Greenblatt, M. Electronic Structure of Sodium Tungsten Bronzes NaWO3 by High-Resolution Angle-Resolved Photoemission Spectroscopy. *Phys. Rev. B* **2007**, *75* (15), 155116. https://doi.org/10.1103/PhysRevB.75.155116.

(2) Mazzola, F.; Chaluvadi, S. K.; Polewczyk, V.; Mondal, D.; Fujii, J.; Rajak, P.; Islam, M.; Ciancio, R.; Barba, L.; Fabrizio, M.; Rossi, G.; Orgiani, P.; Vobornik, I. Disentangling Structural and Electronic Properties in V 2 O 3 Thin Films: A Genuine Nonsymmetry Breaking Mott Transition. *Nano Lett.* **2022**, *22* (14), 5990–5996. https://doi.org/10.1021/acs.nanolett.2c02288.
